# Anti‐*Toxoplasma* activity and chemical compositions of aquatic extract of *Mentha pulegium* L. and *Rubus idaeus* L.: An in vitro study

**DOI:** 10.1002/fsn3.1648

**Published:** 2020-06-08

**Authors:** Hanieh Mohammad Rahimi, Mojdeh Khosravi, Zahra Hesari, Meysam Sharifdini, Hamed Mirjalali, Mohammad Reza Zali

**Affiliations:** ^1^ Foodborne and Waterborne Diseases Research Center Research Institute for Gastroenterology and Liver Diseases Shahid Beheshti University of Medical Sciences Tehran Iran; ^2^ Department of Pharmacy and Pharmaceutical Technology and Parasitology University of Valencia Valencia Spain; ^3^ Department of Pharmaceutics School of Pharmacy Guilan University of Medical Sciences Rasht Iran; ^4^ Department of Medical Parasitology and Mycology School of Medicine Guilan University of Medical Sciences Rasht Iran; ^5^ Gastroenterology and Liver Diseases Research Center Research Institute for Gastroenterology and Liver Diseases Shahid Beheshti University of Medical Sciences Tehran Iran

**Keywords:** herbal medicine, in vitro, *M. pulegium* L., *R. idaeus* L., toxoplasmosis

## Abstract

This study aimed to determine the chemical compositions of crude aquatic extracts of *M. pulegium* L. and *R. idaeus* L., and their anti‐*Toxoplasma* activity. Crude aquatic extraction of aerial parts of *R. idaeus* L. and *M. pulegium* L. was performed. GC‐MS and HTPLC analyses were carried out. MTT assay was performed on Vero cells treated by different concentrations (Log ^−10^ from 10^−1^ to 10^−6^) of the extracts. The anti‐*Toxoplasma* activity of the concentrations was investigated using vital staining. Menthol (99.23%) and limonene (0.227%) were the major compounds of the aquatic extract of *M. pulegium* L. Phytochemical compositions of *R. idaeus* L. were terpenoids, esterols, and flavonoids. The cell toxicity of *M. pulegium* L. was lower than *R. idaeus* L. (CC50 > 10^−2^ versus. ≥ 10^−4^). Aquatic extract of *M. pulegium* L. showed higher anti‐*Toxoplasma* activity (LC50 ≥ 10^−6^) than *R. idaeus* L. (LC50 ≥ 10^−5^). Statistically significant cell toxicity and anti‐*Toxoplasma* activity (*p* < .05) were seen regarding the different concentrations of *R. idaeus* L. and *M. pulegium* L. Both *R. idaeus* L. and *M. pulegium* L. revealed anti‐*Toxoplasma* activities. Cell toxicity of *R. idaeus* L. was significantly higher than *M. pulegium* L. *M. pulegium* L. extract could be more applicable due to its lower cell toxicity.

## Graphical abstract

In the current study, we determined chemical compositions of *M. pulegium* L. and *R. idaeus* L. extracts and evaluated their effects on acute toxoplasmosis that the results were promising.

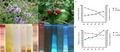

**Abbreviations**
CC50: cell cytotoxicity
GC‐MS: gas chromatography mass spectrometry
HIV: human immunodeficiency virus
LC50: lethal concentration

## 1 INTRODUCTION

Toxoplasmosis caused by an intracellular parasite, *Toxoplasma gondii*, is frequently reported from almost all countries (Montoya & Liesenfeld, 2004; Robert‐Gangneux & Darde, 2012). The infection may occur via vertical transmission from infected mothers, white blood cell (WBC) transfusion, transplantation, consumption of contaminated food and water, and close contact to infected cats (Hide, 2016; Hill & Dubey, 2002, 2016).

Tachyzoite, bradyzoite, and oocyst are the main three forms of *Toxoplasma's* life cycle. Tachyzoites are responsible for the acute phase of toxoplasmosis while bradyzoites are seen in tissue cysts and chronic phase of infection (Montoya & Liesenfeld, 2004). Toxoplasmosis is known as an asymptomatic infection in the chronic phase. Nonetheless, acute toxoplasmosis could be a life‐threatening infection which is mostly reported from immunocompromised patients, particularly HIV/AIDS patients with TCD_4_ counts less than 100/mm^3^ peripheral blood (Ahmadpour et al., 2019; Faucher, Moreau, Zaegel, Franck, & Piarroux, 2011; Safarpour et al., 2020). Currently, a panel of antibiotics is being recommended and widely practiced for both chronic and acute toxoplasmosis (Alday & Doggett, 2017). Accordingly, a combination of sulfadiazine and pyrimethamine is known as a standard antibiotic therapy for toxoplasmosis (Alday & Doggett, 2017; Montazeri et al., 2017). Moreover, clindamycin, pentamidine, atovaquone, and azithromycin are another drugs of choice which are widely prescribed in the clinical practices (Alday & Doggett, 2017). However, low bioavailability, need for a high dosage, and wide side effects including bone marrow suppression, diarrhea, and abdominal pain limit prescription of these drugs (Alday & Doggett, 2017; Darade, Pathak, Sharma, & Patravale, 2018).

During the recent decades, herbal medicine has been explained and practiced as an alternative therapy with low toxicity for a broad range of infective and noninfective diseases (Choi, Gang, & Yun, 2008; Kheirandish et al., 2016; Seo et al., 2019; Yadav & Temjenmongla, 2012; Yeom et al., 2015). *Mentha* belongs to Labaiatae family (Bhat, Maheshwari, Kumar, & Kumar, 2002) and is found all over the world. *Mentha pulegium* L. is a species of this genus that its antimicrobial effects have been evaluated on bacteria and protozoa (Bouyahya et al., 2017; Mahboubi & Haghi, 2008). *Rubus idaeus* L. belongs to *Rubus* genus and Rosaceae family, mainly grows in the north regions of Iran (Moreno‐Medina, Casierra‐Posada, & Cutler, 2018; Nalbandi, Seiiedlou, Hajilou, & Adlipour, 2011). Although many studies investigated the anticancer, anti‐inflammation, and antimicrobial effects of the genus *Rubus* (Krauze‐Baranowska, Glod, et al., 2014; Krauze‐Baranowska, Majdan, et al., 2014; Seo et al., 2019), there is no available data suggesting anti‐parasitic effects of this plant.

In the current study, chemical compositions of crude aquatic extracts of *M. pulegium* L. and *R. idaeus* L. and their anti‐*Toxoplasma* were evaluated.

## 2 MATERIAL AND METHODS

### 2.1 Extract preparation

*R. idaeus* L. and *M. pulegium* L. aerial parts were hand‐picked on same day from a local farm in the northern parts of Iran in August 2016. Plants were air‐dried in 25°C, away from sunshine, with continuous air ventilation until a constant weight obtained. Extraction was performed on powdered dry plant using decoction technique. Briefly, 500 g of plants were subjected to 2 L of distilled water and boiled until the volume decreased to its 1/4. Then, the mixtures were filtered and the extracts were concentrated using indirect heat.

### 2.2 Extract analyses

#### 2.2.1 Gas chromatography (GC)/mass spectrometry (MS)

Crude aquatic extracts of *R. idaeus* L. and *M. pulegium* L. were subjected to a solvent–solvent partitioning with petroleum ether, chloroform, and ethanol. Main components existing in resulted fractions were identified by GC‐MS using a HP‐5ms column (30 m × 0.25 mm, film thickness 0.25 μm; Agilent Technology). Temperature of the column was maintained at 50˚C for 1 min and programmed to 250˚C at a rate of 3˚C per min, and was constant at 250˚C for 20 min. Injector and detector temperatures were 250˚C and 230˚C, respectively. The flow rate of the carrier gas was 1 ml/min. Helium 99.999% was used as the carrier gas with ionization voltage of 70 eV. Mass range was from 40 to 400 u. Identification of the components was performed by comparison of their relative retention time and mass spectra to the standards (Wiley 7 library data of the GC‐MS system).

#### 2.2.2 HPTLC analysis

##### Instruments

Silica gel 60 F254 glass plates (10 × 20 cm with 200 µm thickness HPTLC; Merck), CAMAG automatic thin layer chromatographic (TLC), Sampler 4 (ATS 4), CAMAG automatic developing chamber 2 (ADC 2), CAMAG TLC Visualizer, and winCATS version 1.4.4 software (CAMAG) were used in this study.

##### Solvent extraction

In order to cover a wide range of polarity, 0.1 g of *R. idaeus* L. total extract was subjected to a serial extraction with four organic solvents, starting from an absolutely nonpolar solvent (hexane) followed by gradually increasing polarity solvents (ethyl acetate, chloroform, methanol). Next, acid and alkaline extraction were performed on methanol components by using HCl 0.1 M and NH_3_ 10% v/v. The final extraction of salts was accomplished with chloroform.

##### Chromatographic experiment and phytochemical screening

HPTLC is illustrative for expansion of chromatographic fingerprints to determine major active constituents of medicinal plants. It also presented a more efficient separation of individual secondary metabolites. 10 µl of each sample solutions were applied on the TLC plate using ATS 4 in the form of band (band width: 6 mm, distance between two bands: 9.4 mm). A constant application rate of 150 nl/s was used with the mobile phase of chloroform–methanol (8:2) v/v. The plates were then placed in the mobile phase, and ascending development was performed to a distance of 7 cm. Subsequent to the development, the plates were air‐dried and chromatograms were evaluated with TLC visualizer under 254 nm and under 366nm, and white light.

Qualitative TLC analysis was performed. Accordingly, chloroform–methanol (7:3) was the mobile phase system and silica gel 60 F 254 HPTLC plate was incorporated as stationary phase. Five different reagents including anisaldehyde sulfuric acid, dragendorff, ninhydrin, potassium hydroxide, and sulfuric acid were utilized to describe the secondary metabolic compounds (terpenoids, saponins, alkaloids, amino acids, antraquinones), which were found in extracts. The plate was documented in daylight and at UV 366 nm mode using the photo‐documentation chamber.

### 2.3 *T. Gondii* strain

At the first step, the RH strain of *T. gondii* was kindly supplied by Dr. SJ Seyed Tabaei from intraperitoneal (IP) passages of *Toxoplasma* in female BALB/c mice (8‐ to 10‐week‐old, 20–25 g weight), 3–4 day after IP injection with 1 × 10^7^ of the parasite. All mice were housed in cages under standard laboratory conditions including an average temperature (20–25°C), humidity (60 ± 10%), light (12 hr per day), given drinking water, and regular diet in the animal center of Shahid Beheshti University of Medical Sciences of Tehran, Iran.

The tachyzoites were collected from peritoneal cavity of infected mice. Afterward, the parasites were washed using sterile PBS (pH: 7.4), counted by hemocytometer slide, and 1.5 × 10^6^ tachyzoites were inoculated to the Vero cells, kidney fibroblast from the African green monkey, cultivated in dulbecco's modified eagle's medium (DMEM) supplemented with 10% heat‐inactivated fetal bovine serum (FBS) and 1% penicillin/streptomycin for mass‐cultivation.

### 2.4 Cell culture

Vero cells were used for in vitro assays. In this regard, the cells were cultivated in DMEM medium, supplemented with 10% heat‐inactivated FBS and 1% penicillin and streptomycin. Cultured media were maintained at 37°C in 5% CO_2_. When the cells reach to 80% confluent, sub‐culture was performed.

### 2.5 Cell toxicity assay for *Vero* cell

To evaluate the cell toxicity of the herbs, Vero cells were seeded in 96‐well plates (cell suspensions 2.4 × 10^5^ cell/mL in complete culture medium DMEM and incubated at 37°C in 5% CO_2_ for 48 hr). After 48 hr, serial dilutions 10^−1^ to 10^−6^ of each *R. idaeus* L. and *M. pulegium* L. extract were added and incubated at 37°C in 5% CO_2_ for 2 days. After 48 hr, the cell viability was measured by adding MTT solution (3‐(4,5‐dimethylthiazol‐2‐yl)‐2, 5‐diphenyltetrazolium bromide) to the cultures. A well of Vero cell without treatment was considered as negative control. After 4 hr, the experiment was stopped by DMSO and the results were read at wavelength 590 using an enzyme‐linked immunosorbent assay (ELISA) microplate reader (LX800; Biotec, Winooski, VA, USA). All experiments were performed in duplicate. The nonviable Vero cells were calculated using the following equation (Khosravi et al., 2020), and the 50% cytotoxic concentrations (CC50s) were calculated using the Graph Pad Prism 6.0 software (Graph Pad Software, Inc., San Diego, USA).

$$\text{Viable microorganisms}\;\%=\left[\left(\text{AT}-\text{AB}\right)/\left(\text{AC}-\text{AB}\right)\right]\times 100,$$

Nonviable microorganisms%=100-Viable microorganisms%.

AT is the OD of treated well, AC is the OD of negative control, and AB is the OD of the blank well.

### 2.6 Effect of the plant extracts on tachyzoites of *Toxoplasma*

To evaluate the toxicity of the extracts, 1 × 10^6^ tachyzoites per well of *T. gondii* were added to a 96‐well plate containing DMEM supplemented with 10% FBS without antibiotics; then, serial dilutions 10^−1^ to 10^−6^ of the extracts were added to wells. After 2 hr, 10 µl of *T. gondii* from each well containing different concentrations of the extracts was stained by trypan blue, and the number of alive cells was calculated using hemocytometer slide and optical microscopy. A well containing *T. gondii* without any herbal extract was considered as negative control. All tests performed in duplicate.

$$1-\frac{\text{Test}}{\text{Control}}\times 100.$$

The above equation represents the percentage of the dead tachyzoites. Test: the mean number of alive tachyzoites in each concentration; control: the mean number of alive tachyzoites in control wells.

The lethal concentration (LC) was calculated and the mean 50% (LC50) was estimated from the dose–response curves of *M. pulegium* L. and *R. idaeus* L. different concentrations by using the Graph Pad Prism 6.0 software.

### 2.7 Statistical analysis

Statistical analyses were performed on all data using Graph Pad Prism (version 6.07) software. Differences between test and control groups were analyzed by one‐sample *t* test. *p* < .05 was considered as statistically significant.

## 3 RESULTS

### 3.1 GC‐MS analysis

In all three fractions of *M. pulegium* L., menthol and limonene were identified as two components (Table 1). As a result, menthol was the main component of *M. pulegium* L. (99.23%). Moreover, *R. idaeus* L. was constituted by at least fifteen components with approximately equal percentage while 3‐Decen‐5‐one, 2‐methyl‐ (CAS) and m‐Cymene showed the higher (1.198%) and lower (0.017%) percentages (Table 2).

**TABLE 1:** Main components of *M. pulegium* L. identified by GC‐MS

Peak #	RT*	Compound	Area	%
1	4.2	Limonene	292.00623	0.227
2	7.4	Menthol	1.27583e5	99.233

* Retention time.

**TABLE 2:** Main components of *R. idaeus* L. identified by GC‐MS

Peak #	RT	Compound	Area	%
1	10.76	m‐Cymene	163,664	0.017
2	12.774	Hexachloroethane	3,706,218	0.388
3	17.193	1,2‐Dipentylcyclopropene	1,638,278	0.172
4	20.551	Carvone	4,359,664	0.457
5	22.055	1‐Oxaspiro[4.5]dec−7‐ene, 2,10,10‐trimethyl−6‐methylene‐, trans‐(.+‐.)‐	819,644	0.086
6	22.381	Anethole	320,748	0.034
7	27.201	Tetradecane	2,286,912	0.240
8	31.263	Pentadecane	4,110,038	0.430
9	35.121	Hexadecane	3,327,976	0.349
10	47.925	3‐Decen−5‐one, 2‐methyl‐ (CAS)	11,441,980	1.198
11	49.471	Sulfur	2,341,202	0.245
12	53.763	7,9‐Dihydroxy−5‐methoxy−2‐methyl−1,4‐anthracenedione	503,916	0.053
13	63.808	6 METHYL−2 PHENYLINDOLE	1,018,981	0.107
14	64.621	1‐Methyl−2‐phenylindole	899,925	0.094
15	65.27	2‐Methyl−3‐phenylindole	556,425	0.058

### 3.2 Phytochemistry and HPTLC analyses

**FIGURE 1:**
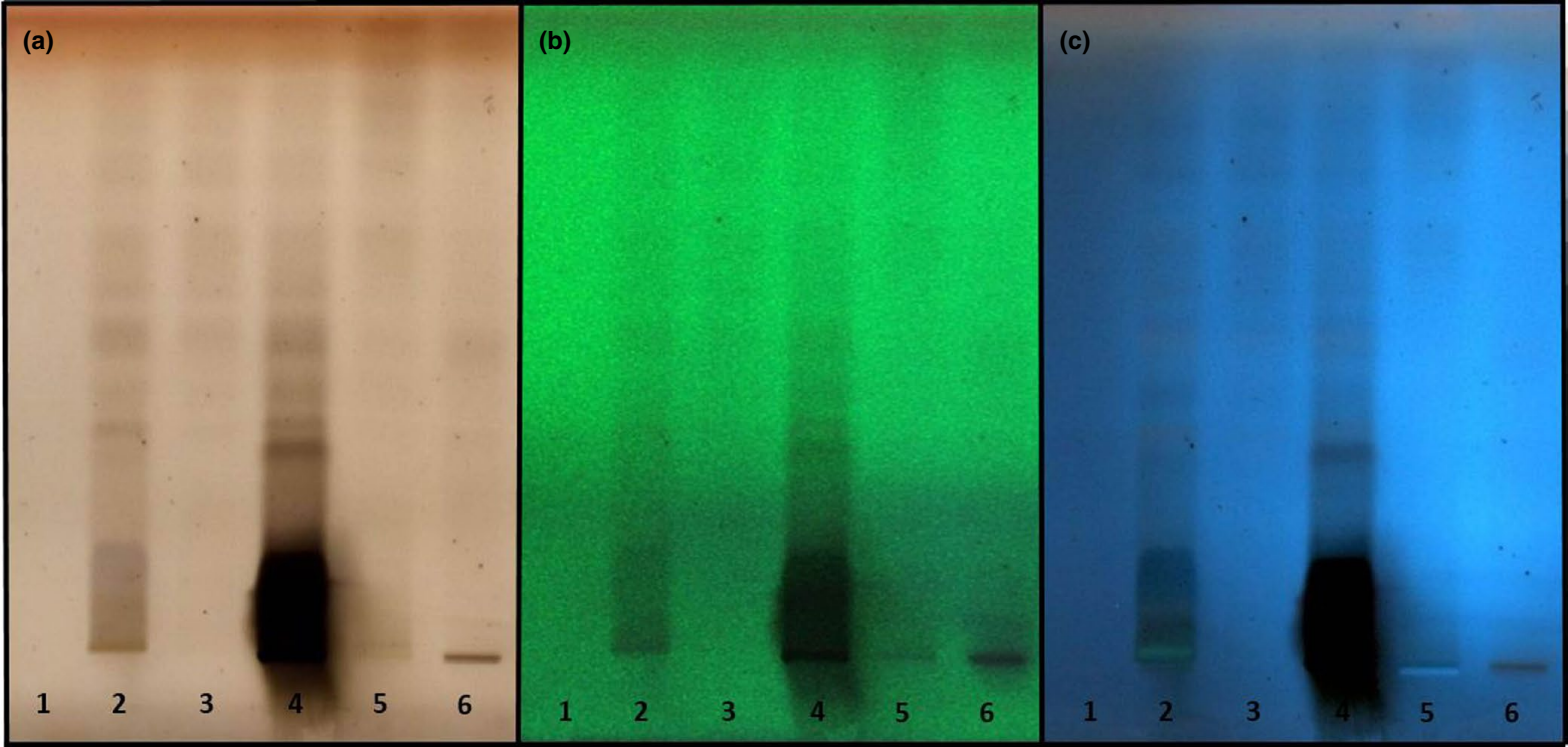
HPTLC analysis of *R. idaeus* L. Total extract was again extracted with: 1‐hexane 2‐ethyl acetate 3‐chloroform 4‐methanol 5‐HCL 6‐NH3. under (a) brown white light (b) green 366 nm and (c) blue 254 nm

**FIGURE 2:**
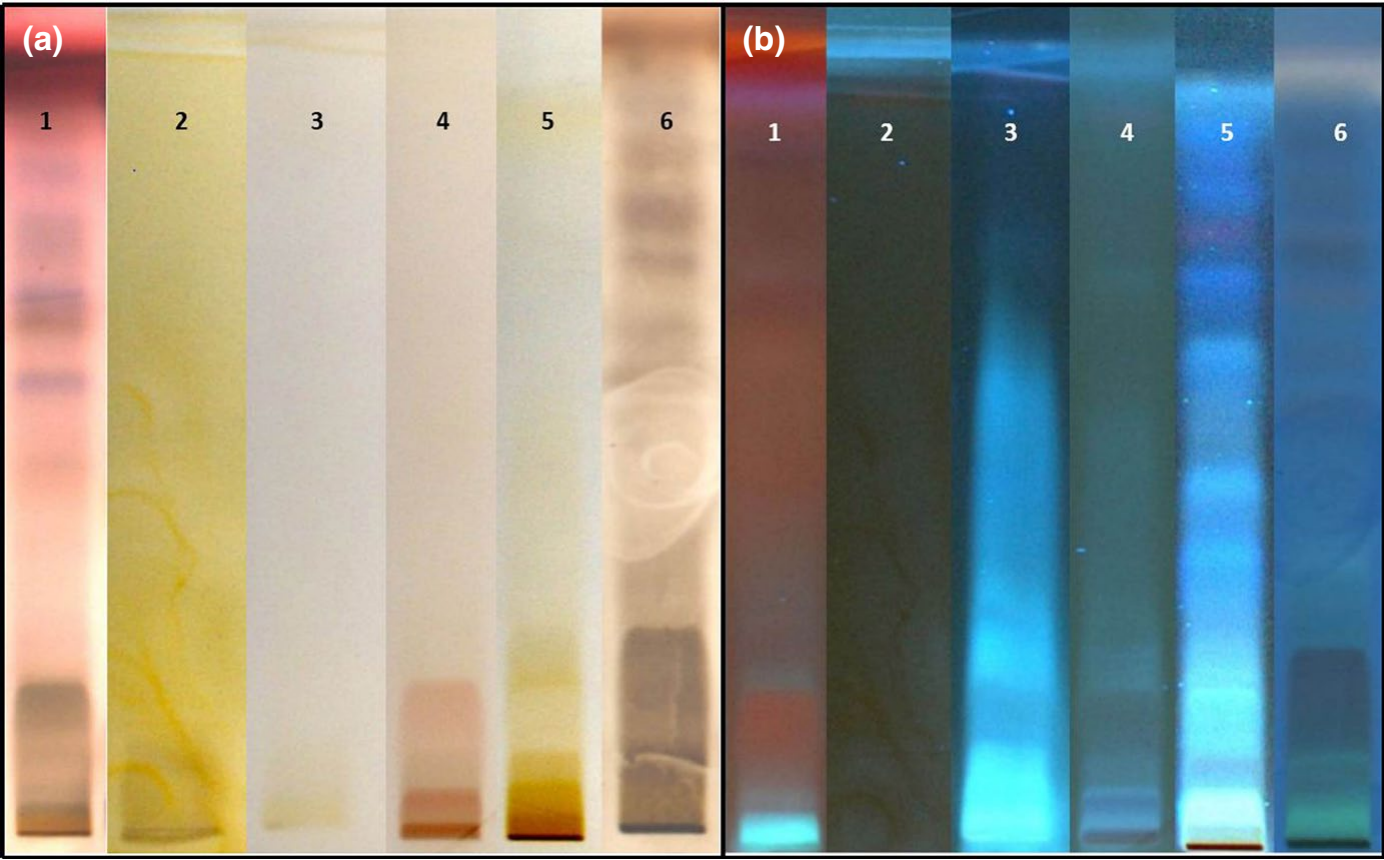
Phytochemical analysis of *R. idaeus* L. extract under (a) visible and (b) UV light with secondary metabolite reagents: 1‐ anisaldehyde sulfuric acid, 2‐ dragendorff`s, 3‐ natural products, 4‐ ninhydrin, 5‐ potassium hydroxide, and 6‐ sulfuric acid

HPTLC presented the fingerprint of *R. idaeus* extract's constituents. Constituents were individualized due to a serial extraction of total extract with hexane, ethyl acetate, chloroform, methanol, HCl, and NH_3_ as specified spots on TLC paper, under white light, and UV light (366 and 254 nm) (Figure 1). Preliminary phytochemical analysis obtained from exposure of *R. idaeus* fingerprint to secondary metabolite reagents (anisaldehyde sulfuric acid, dragendorff`s, ninhydrin, potassium hydroxide, and sulfuric acid) revealed the presence of terpenoids, esterols, and flavonoids based on color zones obtained (Figure 2).

### 3.3 Cell viability of Vero in different concentrations of *M. pulegium* L. and *R. idaeus L*. extracts

To analyze the toxicity of extracts on host cells, we investigated cell viability of Vero cell treated with *M. pulegium* L. and *R. idaeus* L. at the concentrations ranging from 10^−1^ to 10^−6^ using the MTT assay.

#### 3.3.1 *R. idaeus* L

**FIGURE 3:**
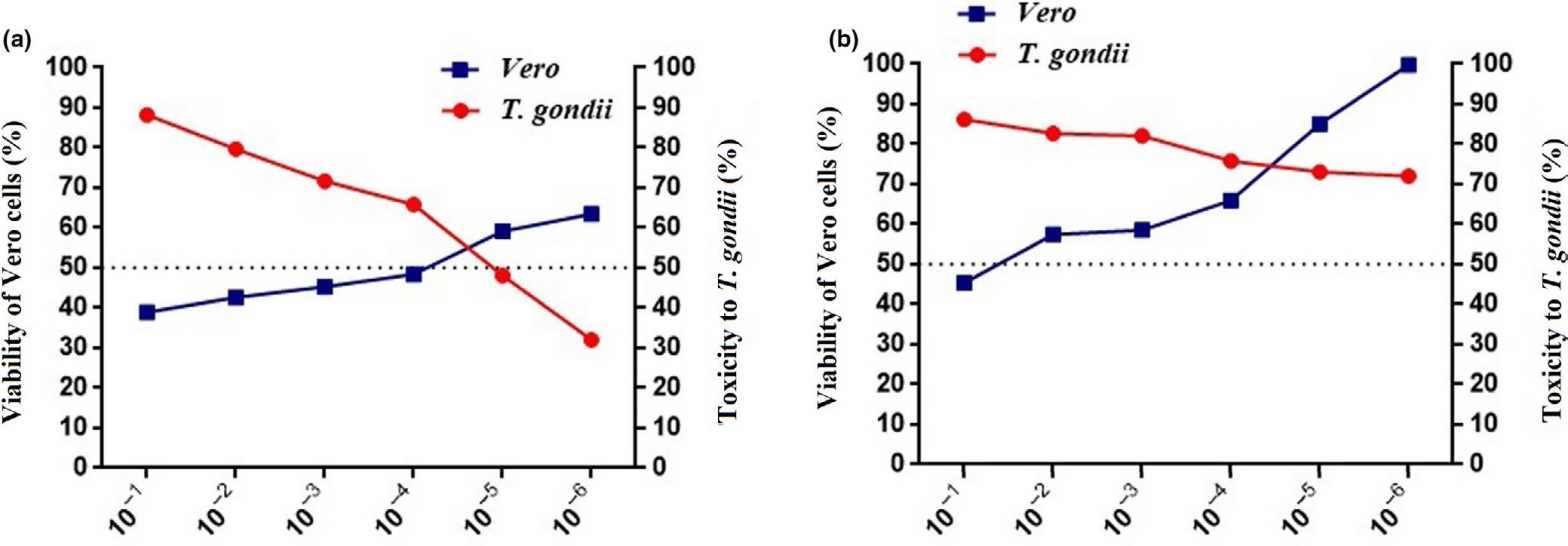
Ratio analyses of (a) *R. idaeus* L. and (b) *M. pulegium* L. show anti‐*Toxoplasma* and cell toxicity of the extracts. CC50 and LC50 for *R. idaeus* L. are at the concentrations more than 10^−4^ and 10^−5^, respectively, while CC50 for *M. pulegium* L. is more than 10^−2^, and LC50 is ≥ 10^−6^

MTT results showed that *R. idaeus* L. extract significantly reduced cell viability of Vero cells in concentrations more than 10^−5^. Accordingly, the results showed that the CC50 of *R. idaeus* L. was about 10^−4^ (CC50 ≥ 10^−4^). The results showed that in the highest concentrations (10^−1^) of the extract, 40% of Vero cells survived while at the lowest concentration (10^−6^), 60% of Vero cells survived (Figure 3a; Table 3).

**TABLE 3:** Viability of Vero cell to different concentrations of *M. pulegium* L. and* R. idaeus* L

Concentrations Log^10^	*M. pulegium* L.		*R. idaeus* L.		***P‐*value** **<0.0001**
	Mean (%) ± *SD*	95% CI	Mean (%) ± *SD*	95% CI	
10^−1^	45.2 ± 0.255	42.913 to 47.487	39.66 ± 0.509	35.086 to 44.234	*
10^−2^	58.18 ± 0.071	57.545 to 58.815	43.595 ± 0.403	39.974 to 47.216	
10^−3^	61 ± 0.636	55.282 to 66.713	45.735 ± 0.417	41.978 to 49.483	
10^−4^	68.655 ± 0.544	63.763 to 73.547	50.225 ± 0.177	48.637 to 51.813	
10^−5^	88.115 ± 0.629	82.461 to 93.769	59.905 ± 0.148	58.571 to 61.239	
10^−6^	98 ± 0.156	96.602 to 99.398	63.865 ± 0.516	59.227 to 68.503	

* statistically significant.

#### 3.3.2 *M. pulegium* L

MTT results of treatment with *M. pulegium* L. showed CC50 higher than 10^−2^ (CC50 > 10^−2^). In addition, apart from the highest concentration (10^−1^), in all other concentrations (lower then 10^−2^), more than 60% of Vero cells were observed alive. Also the results showed that at the lowest concentration (10^−6^), almost 100% of the cells survived (Figure 3b; Table 3).

### 3.4 Effects of *M. pulegium* L. and *R. idaeus* L.extracts on *T. gondii*

To analyze the anti‐*Toxoplasma* effects of each extract, different concentrations of *M. pulegium* L. and *R. idaeus* L. extracts were examined.

#### 3.4.1 *R. idaeus* L

The *R. idaeus* L. extract showed anti‐*Toxoplasma* activity at all concentrations. However, all concentrations more than 10^−5^ were able to kill more than 50% of the parasites. In addition, in the highest concentrations (10^−1^), *R. idaeus* L. extract was toxic for more than 90% of the parasites. Therefore, LC50 of *R. idaeus* L. was ≥ 10^−5^ (LC50 ≥ 10^−5^) (*P‐*value < 0.05) (Figure 3a; Table 4).

**TABLE 4:** Anti‐*Toxoplasma* activity of different concentrations of *M. pulegium* L., and *R. idaeus* L., and ratio values (anti‐parasite activity per Vero cell viability)

Concentrations (Log 10^−1^)	*M. pulegium* L.			*R. idaeus* L.			*P‐*value <0.0001
	Mean (%) ± *SD*	95% CI	Ratio	Mean (%) ± *SD*	95% CI	Ratio	
10^−1^	86.65 ± 0.636	80.932 to 92.368	1.91	89.31 ± 0.410	85.625 to 92.995	2.25	**
10^−2^	83.1 ± 0.424	79.288 to 86.912	1.42	80.1 ± 0.410	76.415 to 83.785	1.83	
10^−3^	83 ± 0.424	79.188 to 86.812	1.36	73.55 ± 0.636	67.832 to 79.268	1.61	
10^−4^	74.55 ± 0.354	71.373 to 77.727	1.08*	70.755 ± 0.346	67.642 to 73.868	1.41	
10^−5^	72.91 ± 0.269	70.496 to 75.324	0.82	51.655 ± 0.502	47.144 to 56.166	0.86*	
10^−6^	70.99 ± 0.014	70.863 to 71.117	1.38	32.33 ± 0.467	32.330 to 36.523	0.50	

* The ratios closer to 1 show the best concentration of drugs with highest anti‐*Toxoplasma* activity and lowest Vero toxicity.

** Statistically significant.

#### 3.4.2 *M. pulegium* L

The *M. pulegium* L. extract showed high anti‐*Toxoplasma* activity (killed more than 70% of the parasites) at all concentrations with *P‐*value < 0.05 (LC50 ≥ 10^−6^) (Figure 3b; Table 4).

### 3.5 Ratio analysis

In order to calculate the best concentration with highest anti‐*Toxoplasma* activity and lowest cell toxicity, ratio analysis was performed. The value closer to 1 considered as the recommended value. Accordingly, 10^−4^ with ratio 1.08 was the suggested concentration with highest and lowest anti‐*Toxoplasma* and cell toxicity, respectively, for *M. pulegium* L. The concentration 10^−5^ of *R. idaeus* L. with ratio 0.86 seems to be the best concentration.

## 4 DISCUSSION

During recent years, interest on the traditional drugs, particularly herbal medicine, is being dramatically increased (Kheirandish et al., 2016). It seems that lower side effects and costs are the main reasons of this interest. Plenty of studies have practiced different herbal extracts on both helminths (Dejani et al., 2014; Yadav & Tangpu, 2008, 2012; Yadav & Temjenmongla, 2012) and protozoa (Dyab, Yones, Ibraheim, & Hassan, 2016; Kheirandish et al., 2016; Mirzaalizadeh et al., 2018; Ribeiro et al., 2014) that the results of most of them were promising. Among parasites, although most of experiments were carried out on *Leishmania* spp. (Bouyahya et al., 2017; Kheirandish et al., 2016; Ribeiro et al., 2014), a couple of studies evaluated the anti‐*Toxoplasma* effects of herbal extracts using in vitro and in vivo studies (Al Nasr, Ahmed, Pullishery, El‐Ashram, & Ramaiah, 2016).

Acute toxoplasmosis could be a fatal infection in immunocompromised patients (Montoya & Liesenfeld, 2004). Although there is a list of drugs of choice for both acute and chronic toxoplasmosis, the main limitations of the current antibiotics are low bioavailability, and side effects (Alday & Doggett, 2017; Darade et al., 2018). In the current study, the chemical compositions of *M. pulegium* L. and *R. idaeus* L. from the local farms in the north of Iran were determined and the cell toxicity and anti*‐Toxoplasma* activity of them were evaluated.

The chemical compositions of the essential oil of *M. pulegium* L. were previously analyzed by Mahboubi and Haghi (2008) who reported piperitone, piperitenone, α‐terpineol, and pulegone as the main components. In this line, although studies showed the presence of piperitone, piperitenone, pulegone, and isomenthone/neoisomenthol as the major oil components of the *M. pulegium* L. essential oil (Bouyahya et al., 2017; Mahboubi & Haghi, 2008), there are no data about the components of the aquatic extract of this plant. In the current study, menthol (99.23%) and limonene (0.227%) were the main compounds of the aquatic extract of *M. pulegium* L. It seems that differences during the extract preparation are the main reason for different extracted compounds in our study in comparison to the others.

However, the antimicrobial effects of *M. pulegium* L. and its compounds have been widely practiced. Mahboubi and Haghi (2008) showed that the *M. pulegium* L. essential oil extract had significant antimicrobial effects, particularly on the Gram‐positive bacteria. In studies conducted by Dejani et al. (2014) and Zaia et al., (2016), the anti‐*Schistosoma* (*S. mansoni*) and anti‐inflammatory effects of crude ethanol extract and menthol from *M. piperita* L., respectively, were investigated that the findings were promising. In another study by Bouyahya et al. (2017)*,* pulegone and menthone were characterized as the major compounds of *M. pulegium* which showed anti‐*Leishmania* activity at the concentrations from 0.875 to 10 µl/ml. In the current study, the extract of *M. pulegium* L. showed cell toxicity at the concentration > log 10^−2^ which suggests a low cell toxicity at the high concentration. Furthermore, all dilutions (10^−6^ to 10^−1^) showed anti‐*Toxoplasma* activity (more than 70% of the parasite) suggesting high anti‐*Toxoplasma* activity beside low cell toxicity at the high concentrations.

Iranian genotypes of *R. idaeus* L. (red raspberry) grow in the northern provinces of the country (Nalbandi et al., 2011). Although chemical compositions and antimicrobial activity of the Iranian strains of this plant have not been investigated, European genotypes of *R. idaeus* L. are known as a traditional herbal medicine in the Eastern Europe (Krauze‐Baranowska, Glod, et al., 2014). Velicanski, Cvetkovic, and Markov (2012) investigated the antimicrobial activity of both fruit and pomace extracts of *R. idaeus* L., and showed antimicrobial activity of the extracts on *Pseudomonas aeruginosa*, *Bacillus cereus*, *Staphylococcus aureus*, and *S. saprophyticus* while *Escherichia coli* was the most resistant bacterium. In the study conducted by Bobinaitė, Viškelis, Šarkinas, and Venskutonis (2013), the contents of fruit, pulp, and marc extractions of raspberry in methanol and acetone solvents were evaluated that despite of differences in antimicrobial activity regarding parts of raspberry and solvents, the results were promising. Moreover, Krauze‐Baranowska, Glod, et al. (2014) revealed that ellagic acid and sanguiin were the predominant compounds of the young shoots of *R. idaeus* L. In addition, they showed antioxidant, antimicrobial (strongly against *Corynebacterium diphtheria*), and cytotoxicity activities (against HeLa and HL‐60 cells, but not on the fibroblastic cells) of the plant.

In the current study for the first time, phytochemical compositions of Iranian genotypes of *R. idaeus* L. were characterized and terpenoids, esterols, and flavonoids were described. Although many studies evaluated the antimicrobial activity of this plant, there are no data on the anti‐parasitic activity of *R. idaeus* L. However, our findings showed LC50 ≥ 10^−5^ against *Toxoplasma* while CC50 ≤ 10^−4^ was observed for Vero cell. The high cell toxicity of *R. idaeus* L. in this study is in line with previous studies representing cytotoxicity of the extracts of this plant on HeLa and HL‐60 cell which are cancer cell lines.

## 5 CONCLUSION

According to our findings, both *R. idaeus* L. and *M. pulegium* L. revealed anti‐*Toxoplasma* activity. In addition, cytotoxicity activity of *R. idaeus* L. was significantly higher than that in *M. pulegium* L. Therefore, although anti‐*Toxoplasma* activity of both *R. idaeus* L. and *M. pulegium* L. extracts might be promising, our results showed that *M. pulegium* L. extract could be more applicable due to its lower cell toxicity in higher concentration in comparison to *R. idaeus* L.

## CONFLICT OF INTEREST

All authors of this manuscript declare that we have seen and approved the submitted version of this manuscript.

## AUTHOR CONTRIBUTIONS

HM: conceived and designed the experiments. HMR MK ZH performed the experiments. HM ZH analyzed the data. MS provided the sample. MRZ contributed reagents/materials/analysis tools/positive samples. HM HMR wrote the paper. All authors read and approved the final version of the manuscript.

## ETHICAL APPROVAL

All procedures performed in this study were in accordance with the ethical standards (IR.SBMU.RIGLD.REC.1398.034) released by the Ethical Review Committee of the Research Institute for Gastroenterology and Liver Diseases, Shahid Beheshti University of Medical Sciences, Tehran, Iran.

## INFORMED CONSENT

Not applicable.

## Data Availability

The data associated with this manuscript are mentioned throughout the manuscript.

## References

[fsn31648-bib-0001] Ahmadpour, E. , Pishkarie‐Asl, R. , Spotin, A. , Samadi Kafil, H. , Didarlu, H. , Azadi, Y. , & Barac, A. (2019). Sero‐molecular evaluation of *Toxoplasma gondii* infection among HIV‐positive patients. Transactions of the Royal Society of Tropical Medicine and Hygiene, 113(12), 771–775. 10.1093/trstmh/trz082 31495900

[fsn31648-bib-0002] Al Nasr, I. , Ahmed, F. , Pullishery, F. , El‐Ashram, S. , & Ramaiah, V. V. (2016). Toxoplasmosis and anti‐*Toxoplasma* effects of medicinal plant extracts‐A mini‐review. Asian Pacific Journal of Tropical Medicine, 9(8), 730–734. 10.1016/j.apjtm.2016.06.012 27569880

[fsn31648-bib-0003] Alday, P. H. , & Doggett, J. S. (2017). Drugs in development for toxoplasmosis: Advances, challenges, and current status. Drug Design, Development and Therapy, 11, 273–293. 10.2147/DDDT.S60973 PMC5279849 28182168

[fsn31648-bib-0004] Bhat, S. , Maheshwari, P. , Kumar, S. , & Kumar, A. (2002). *Mentha* species: In vitro regeneration and genetic transformation. Molecular Biology Today, 3(1), 11–23.

[fsn31648-bib-0005] Bobinaitė, R. , Viškelis, P. , Šarkinas, A. , & Venskutonis, P. R. (2013). Phytochemical composition, antioxidant and antimicrobial properties of raspberry fruit, pulp, and marc extracts. CyTA ‐ Journal of Food, 11(4), 334–342. 10.1080/19476337.2013.766265

[fsn31648-bib-0006] Bouyahya, A. , Et‐Touys, A. , Bakri, Y. , Talbaui, A. , Fellah, H. , Abrini, J. , & Dakka, N. (2017). Chemical composition of *Mentha pulegium* and *Rosmarinus officinalis* essential oils and their antileishmanial, antibacterial and antioxidant activities. Microbial Pathogenesis, 111, 41–49. 10.1016/j.micpath.2017.08.015 28821401

[fsn31648-bib-0007] Choi, K. M. , Gang, J. , & Yun, J. (2008). Anti‐*Toxoplasma gondii* RH strain activity of herbal extracts used in traditional medicine. International Journal of Antimicrobial Agents, 32(4), 360–362. 10.1016/j.ijantimicag.2008.04.012 18619816

[fsn31648-bib-0008] Darade, A. , Pathak, S. , Sharma, S. , & Patravale, V. (2018). Atovaquone oral bioavailability enhancement using electrospraying technology. European Journal of Pharmaceutical Sciences, 111, 195–204. 10.1016/j.ejps.2017.09.051 28974387

[fsn31648-bib-0009] Dejani, N. N. , Souza, L. C. , Oliveira, S. R. , Neris, D. M. , Rodolpho, J. M. , Correia, R. O. , … Anibal, F. F. (2014). Immunological and parasitological parameters in *Schistosoma mansoni*‐infected mice treated with crude extract from the leaves of *Mentha x piperita* L. Immunobiology, 219(8), 627–632. 10.1016/j.imbio.2014.03.015 24767421

[fsn31648-bib-0010] Dyab, A. K. , Yones, D. A. , Ibraheim, Z. Z. , & Hassan, T. M. (2016). Anti‐giardial therapeutic potential of dichloromethane extracts of *Zingiber officinale* and *Curcuma longa* in vitro and in vivo. Parasitology Research, 115(7), 2637–2645. 10.1007/s00436-016-5010-9 26984104

[fsn31648-bib-0011] Faucher, B. , Moreau, J. , Zaegel, O. , Franck, J. , & Piarroux, R. (2011). Failure of conventional treatment with pyrimethamine and sulfadiazine for secondary prophylaxis of cerebral toxoplasmosis in a patient with AIDS. Journal of Antimicrobial Chemotherapy, 66(7), 1654–1656. 10.1093/jac/dkr147 21459896

[fsn31648-bib-0012] Hide, G. (2016). Role of vertical transmission of *Toxoplasma gondii* in prevalence of infection. Expert Review of Anti‐infective Therapy, 14(3), 335–344. 10.1586/14787210.2016.1146131 26807498

[fsn31648-bib-0013] Hill, D. , & Dubey, J. P. (2002). *Toxoplasma gondii*: Transmission, diagnosis and prevention. Clinical Microbiology and Infection, 8(10), 634–640. 10.1046/j.1469-0691.2002.00485.x 12390281

[fsn31648-bib-0014] Hill, D. E. , & Dubey, J. P. (2016). *Toxoplasma gondii* as a parasite in food: Analysis and control. Microbiology Spectrum, 4(4). 10.1128/microbiolspec.PFS-0011-2015 27726776

[fsn31648-bib-0015] Kheirandish, F. , Delfan, B. , Mahmoudvand, H. , Moradi, N. , Ezatpour, B. , Ebrahimzadeh, F. , & Rashidipour, M. (2016). Antileishmanial, antioxidant, and cytotoxic activities of *Quercus infectoria* Olivier extract. Biomedicine and Pharmacotherapy, 82, 208–215. 10.1016/j.biopha.2016.04.040 27470357

[fsn31648-bib-0016] Khosravi, M. , Mohammad Rahimi, H. , Doroud, D. , Mirsamadi, E. S. , Mirjalali, H. , & Zali, M. R. (2020). In vitro evaluation of mannosylated paromomycin‐loaded solid lipid nanoparticles on acute toxoplasmosis. Frontiers in Cellular and Infection Microbiology, 10, 33 10.3389/fcimb.2020.00033 32117807 PMC7031658

[fsn31648-bib-0017] Krauze‐Baranowska, M. , Glod, D. , Kula, M. , Majdan, M. , Halasa, R. , Matkowski, A. , … Kawiak, A. (2014). Chemical composition and biological activity of *Rubus idaeus* shoots–a traditional herbal remedy of Eastern Europe. BMC Complementary and Alternative Medicine, 14, 480 10.1186/1472-6882-14-480 25496130 PMC4295307

[fsn31648-bib-0018] Krauze‐Baranowska, M. , Majdan, M. , Halasa, R. , Glod, D. , Kula, M. , Fecka, I. , & Orzel, A. (2014). The antimicrobial activity of fruits from some cultivar varieties of *Rubus idaeus* and *Rubus occidentalis* . Food Function, 5(10), 2536–2541. 10.1039/C4FO00129J 25131001

[fsn31648-bib-0019] Mahboubi, M. , & Haghi, G. (2008). Antimicrobial activity and chemical composition of *Mentha pulegium* L. essential oil. Journal of Ethnopharmacology, 119(2), 325–327. 10.1016/j.jep.2008.07.023 18703127

[fsn31648-bib-0020] Mirzaalizadeh, B. , Sharif, M. , Daryani, A. , Ebrahimzadeh, M. A. , Zargari, M. , Sarvi, S. , … Montazeri, M. (2018). Effects of aloe vera and eucalyptus methanolic extracts on experimental toxoplasmosis in vitro and in vivo. Experimental Parasitology, 192, 6–11. 10.1016/j.exppara.2018.07.010 30031121

[fsn31648-bib-0021] Montazeri, M. , Sharif, M. , Sarvi, S. , Mehrzadi, S. , Ahmadpour, E. , & Daryani, A. (2017). A systematic review of in vitro and in vivo activities of anti‐*Toxoplasma* drugs and compounds (2006–2016). Frontiers in Microbiology, 8, 25 10.3389/fmicb.2017.00025 28163699 PMC5247447

[fsn31648-bib-0022] Montoya, J. G. , & Liesenfeld, O. (2004). Toxoplasmosis. Lancet (London, England), 363(9425), 1965–1976. 10.1016/S0140-6736(04)16412-X 15194258

[fsn31648-bib-0023] Moreno‐Medina, B. L. , Casierra‐Posada, F. , & Cutler, J. (2018). Phytochemical composition and potential use of *Rubus* species. Gesunde Pflanzen, 70(2), 65–74. 10.1007/s10343-018-0416-1

[fsn31648-bib-0024] Nalbandi, H. , Seiiedlou, S. , Hajilou, J. , & Adlipour, M. (2011). Some post‐harvest properties of Iranian genotype of raspberry (*Rubus ideaus* L.). Australian Journal of Agricultural Engineering, 2(6), 155.

[fsn31648-bib-0025] Ribeiro, T. G. , Chavez‐Fumagalli, M. A. , Valadares, D. G. , Franca, J. R. , Lage, P. S. , Duarte, M. C. , … Castilho, R. O. (2014). Antileishmanial activity and cytotoxicity of Brazilian plants. Experimental Parasitology, 143, 60–68. 10.1016/j.exppara.2014.05.004 24846006

[fsn31648-bib-0026] Robert‐Gangneux, F. , & Darde, M. L. (2012). Epidemiology of and diagnostic strategies for toxoplasmosis. Clinical Microbiology Reviews, 25(2), 264–296. 10.1128/CMR.05013-11 22491772 PMC3346298

[fsn31648-bib-0027] Safarpour, H. , Cevik, M. , Zarean, M. , Barac, A. , Hatam‐Nahavandi, K. , Rahimi, M. T. , … Ahmadpour, E. (2020). Global status of *Toxoplasma gondii* infection and associated risk factors in people living with HIV: A systematic review and meta‐analysis. AIDS. 34(3), 469–474. 10.1097/QAD.0000000000002424 31714356

[fsn31648-bib-0028] Seo, K. H. , Lee, J. Y. , Park, J. Y. , Jang, G. Y. , Kim, H. D. , Lee, Y. S. , & Kim, D. H. (2019). Differences in anti‐inflammatory effect of immature and mature of *Rubus coreanus* fruits on LPS‐induced RAW 264.7 macrophages via NF‐kappaB signal pathways. BMC Complement Alternative Medicine, 19(1), 89 10.1186/s12906-019-2496-6 PMC6485102 31023273

[fsn31648-bib-0029] Velicanski, A. S. , Cvetkovic, D. D. , & Markov, S. L. (2012). Screening of antibacterial activity of raspberry (*Rubus idaeus* L.) fruit and pomace extracts. Acta Periodica Technologica, 2012(43), 305–313. 10.2298/APT1243305V

[fsn31648-bib-0030] Yadav, A. K. , & Tangpu, V. (2008). Anticestodal activity of *Adhatoda vasica* extract against *Hymenolepis diminuta* infections in rats. Journal of Ethnopharmacology, 119(2), 322–324. 10.1016/j.jep.2008.07.012 18691645

[fsn31648-bib-0031] Yadav, A. K. , & Tangpu, V. (2012). Anthelmintic activity of ripe fruit extract of *Solanum myriacanthum* Dunal (Solanaceae) against experimentally induced *Hymenolepis diminuta* (Cestoda) infections in rats. Parasitology Research, 110(2), 1047–1053. 10.1007/s00436-011-2596-9 21842379

[fsn31648-bib-0032] Yadav, A. K. , & Temjenmongla. (2012). In vivo anthelmintic activity of *Clerodendrum colebrookianum* Walp., a traditionally used taenicidal plant in Northeast India. Parasitology Research, 111(4), 1841–1846. 10.1007/s00436-012-2908-8 22476567

[fsn31648-bib-0033] Yeom, M. , Kim, J. H. , Min, J. H. , Hwang, M. K. , Jung, H. S. , & Sohn, Y. (2015). *Xanthii fructus* inhibits inflammatory responses in LPS‐stimulated RAW 264.7 macrophages through suppressing NF‐kappaB and JNK/p38 MAPK. Journal of Ethnopharmacology, 176, 394–401. 10.1016/j.jep.2015.11.020 26560439

[fsn31648-bib-0034] Zaia, M. G. , Cagnazzo, T. , Feitosa, K. A. , Soares, E. G. , Faccioli, L. H. , Allegretti, S. M. , … Anibal Fde, F. (2016). Anti‐inflammatory properties of menthol and menthone in *Schistosoma mansoni* infection. Frontiers in Pharmacology, 7, 170 10.3389/fphar.2016.00170 27378927 PMC4911957

